# Molecular determinants of α-synuclein toxicity and aggregation revealed by deep mutagenesis

**DOI:** 10.1042/BST20250295

**Published:** 2026-11-03

**Authors:** Daeun Noh, Robert W. Newberry

**Affiliations:** Department of Chemistry, The University of Texas at Austin, 105 E 24th St. Austin, TX, 78712, U.S.A.

**Keywords:** alpha synuclein, deep mutational scanning, protein aggregation, protein misfolding

## Abstract

The abundant neuronal protein α-synuclein is a key driver of Parkinson’s and related diseases. α-Synuclein adopts a wide variety of different conformational states, but it is often unclear which of these species is responsible for its various behaviors, complicating efforts to combat pathologic misfolding of α-synuclein. Whereas conventional approaches struggle to provide direct, high-resolution insight into these structure–activity relationships, deep mutational scanning (DMS) has recently emerged as a powerful approach to probe the structural basis of protein activity and identify the molecular determinants of pathology. In the present review, we highlight insights from recent DMS studies of α-synuclein, which have implicated membrane binding as an important contribution to its toxicity and aggregation. Further, experiments in different cell types reveal distinct determinants of aggregation, highlighting the role of the cellular environment in shaping the conformational landscape of α-synuclein. We therefore expect that DMS will fill an important gap in the characterization of protein structure–activity relationships, particularly for conformationally dynamic proteins, including those implicated in misfolding diseases.

## Introduction

### α-Synuclein misfolding is pathogenic and heterogeneous

α-Synuclein is a key component of Lewy bodies, the pathological hallmark of Parkinson’s disease (PD) [1], and its misfolding and aggregation are central to disease pathogenesis [2–7]. This small (140-residue, 14.5-kDa), intrinsically disordered protein is enriched at presynaptic terminals [8]. Its primary sequence can be roughly divided into three regions (Figure 1A): an N-terminal region (residues 1–60) containing seven concatenated 11-residue amphiphilic repeats that mediate membrane binding [9]; the hydrophobic, amyloid-prone non-amyloid β component (NAC) domain (residues 61–94) that drives aggregation [10,11]; and an acidic, conformationally dynamic C-terminal region (residues 95–140). This partition is only approximate, as the functional boundaries between regions are not sharply defined. For example, the amphiphilic repeats extend from the N-terminal region into the hydrophobic NAC region, and membrane binding engages the N terminus together with the N-terminal portion of the NAC so that the lipid-bound amphiphilic helix can span residues approximately 1–100 [12–14]. Similarly, though short segments in the hydrophobic NAC region (e.g., residues 68–78) are generally the most amyloidogenic [15], large sections of the N-terminal and hydrophobic NAC domains can be incorporated into misfolded structures (Figure 1B–F) [16,17].

**Figure 1:**
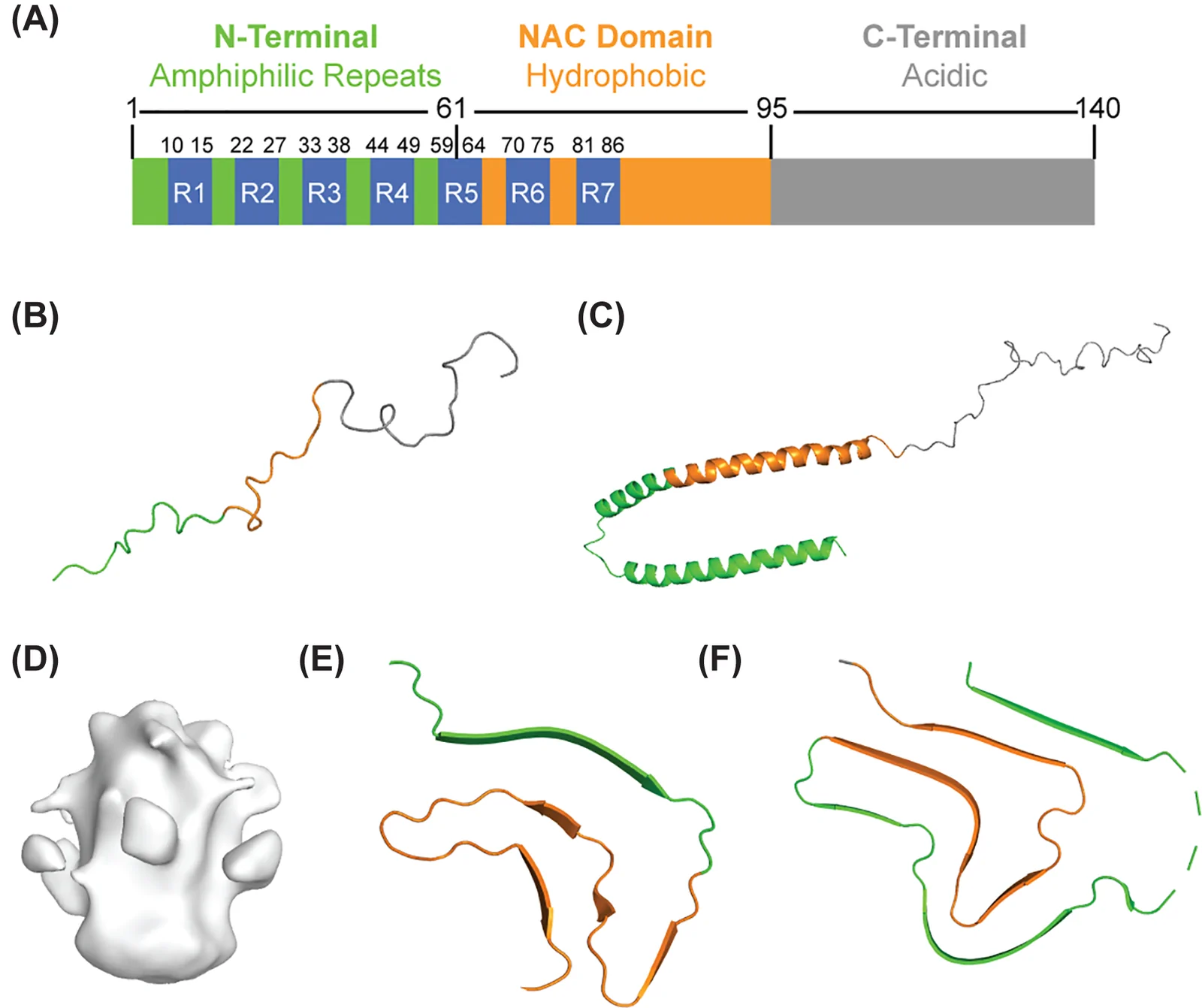
Structure of α-synuclein (**A**) Primary sequence of α-synuclein. R1–R7 denote the KTKEGV motifs of the imperfect repeats within the membrane-binding region. (**B**) Hypothetical model of intrinsically disordered monomeric α-synuclein. (**C**) Nuclear magnetic resonance (NMR)-derived model of micelle-bound monomeric α-synuclein in a broken-helix conformation (PDB 1XQ8) [12]. Data from EPR [13], solid-state NMR [14], and Förster resonance energy transfer (FRET) [18] indicate that α-synuclein likely adopts an unbroken helix in the presence of other model membranes such as bilayer vesicles. (**D**) Cryo-EM model of oligomeric α-synuclein (EMD: 16466) [19]. (**E**) Cryo-EM model of fibrillar α-synuclein (PDB: 6H6B) [20]. (**F**) Cryo-EM structure of lipidic α-synuclein amyloid fibrils (PDB: 8A4L) [21].

One of the most challenging problems plaguing efforts to understand the role of α-synuclein in pathology is its remarkable conformational diversity. In solution, α-synuclein is predominantly disordered [22,23], but it can form amphiphilic α-helices upon membrane binding [9,12–14,24], oligomeric assemblies with variable secondary structure [19,25–29], cross-β amyloid fibrils [21,30–33], and phase-separated condensates that can mature into aggregated species [34,35]. These conformations differ in size, secondary structure, membrane affinity, seeding activity, and toxicity [17,36,37]. Importantly, all of these structural insights are derived from studies of purified material in a cell-free context. This conformational diversity is central to disease [17,38], as distinct conformations are observed in patients afflicted by different synucleinopathies [33], and conformationally distinct preparations of recombinant α-synuclein induce distinct phenotypes in disease models [39–42]. Given the remarkable diversity of conformational states, including the disordered monomer, membrane-bound helix, soluble oligomers, amyloid fibrils, and condensate-derived aggregates, it has been difficult to understand the molecular-level events that cause the protein to become toxic and pathologic. Furthermore, it remains unclear which of these states drive specific cellular phenotypes, which is important for developing therapeutic strategies. For example, interventions that reduce amyloid formation might not suppress membrane-associated toxicity, whereas strategies that weaken membrane binding might alter physiological vesicle trafficking [43–45].

A central barrier to addressing these challenges is the difficulty in probing α-synuclein structure in living disease models, which could enable structure–function relationships to be established directly. Most structural studies are performed *in vitro* (i.e., cell-free), which can provide high-resolution mechanistic information; however, purified proteins, model membranes, and simplified buffers cannot fully reproduce molecular crowding, organelle membranes, proteostasis systems, post-translational modifications, and binding partners that can shape the folding landscape. Conversely, measurements in living cells can preserve biological context but often report low-resolution phenotypes such as growth defects, puncta formation, or fluorescence redistribution. In-cell NMR [46,47], electron tomography [48,49], and advanced fluorescence approaches [50–53] have provided important insights [53,54], but they remain difficult to apply broadly across different structural states, concentration regimes, and biological models. Thus, understanding α-synuclein misfolding requires methods that directly connect cellular phenotype to molecular structure and can be applied within native biological contexts.

### Mutagenesis: a key approach to probe structure–activity relationships

Mutational perturbation is one powerful method to bridge this gap. If an amino-acid substitution affects the activity of the protein, the affected residues and its physicochemical properties are implicated in that activity, establishing key features of the structure–activity relationship. Importantly, substitutions that disrupt or stabilize particular conformations can implicate those conformations in associated phenotypes, even without visualizing the conformer directly. Early mutagenic studies of α-synuclein focused on variants discovered in families afflicted by inherited, early-onset PD, which provided some of the first causal links between α-synuclein and neurodegeneration [55,56]. Subsequent genetic studies have discovered additional variants associated with familial PD [57–59], and variants associated with sporadic PD continue to be reported [60]. Most of these residues cluster in the N terminal region, specifically between residues 30 and 55, implicating this region as a key determinant of pathology. However, these substitutions can have divergent effects on the same molecular process, which can be muddy or confound the interpretation of the underlying molecular determinants. For example, the PD-associated mutations A30P and A53T can have quantitatively different effects on both membrane binding and fibrillization relative to WT [61–63], making it difficult to understand how these processes contribute to disease etiology.

Though these natural variants have been widely exploited to develop disease models [64–67] and observe disruptions in disease-associated physiological processes, known natural variants only sparsely sample the full α-synuclein sequence, further limiting insights into the conformational determinants of toxicity. As a result, biochemists have engineered a wide variety of mutations into α-synuclein to test specific molecular hypotheses [68]. For example, substitutions can be engineered to either mimic key post-translational modifications (e.g., phosphomimetic S/T/Y→D/E substitutions) [69–71] or suppress them (e.g., ubiquitylation-disrupting K→R substitutions or M→L substitutions that prevent thioether oxidation) [72,73]. Engineered mutations have also been exploited to probe the functional roles of intermolecular interactions such as those with metal ions [74,75] or lipid membranes [18,76,77]. Moreover, because the synuclein sequence is repetitive, additional insights can come from combining related mutations; for example, the Selkoe and Dettmer labs have designed a 3K variant (E35K/E46K/E61K) that supercharges the effect of a familial mutation on α-synuclein aggregation, resulting in monomer accumulation at membranes, inclusion formation, and motor impairment in mice [78–80]. Mutagenesis has also been exploited to probe interfaces in pathological protein aggregates; for example, mutations that either disrupt or stabilize amyloid folds have been exploited to probe the formation of these species in different biological contexts [69,81–84].

Interpreting the effects of individual mutations can be challenging, in part because each mutation inevitably perturbs multiple molecular properties. For example, phosphomimetic mutations not only change the local charge but can also alter local conformational propensities for α-helix or β-sheet. Without complementary measurements, these effects can be difficult to deconvolute. Additional insights can therefore be gleaned from efforts to increase the mutational coverage, which provides opportunities to test the same hypothesis from multiple different perturbations and to identify cross-cutting trends. For example, Volles and Lansbury performed a random mutagenesis screen that implicated membrane binding as a consistent determinant of toxicity in yeast [85]. Subsequently, Koo et al. performed extensive rational mutagenesis on α-synuclein, including a partial proline scan, which highlighted a role of the hydrophobic NAC region in amyloid nucleation and elongation [86,87]. Burré et al. later generated a panel of 26 engineered α-synuclein variants in order to probe a variety of synuclein-related processes, including physiological chaperoning of SNARE-complex formation, as well as neurotoxicity in cellular models. Their data suggest that physiological and pathological processes are governed by nonoverlapping determinants [88]. Together, these studies highlight the power of systematic mutagenesis to help develop coherent models of protein activity.

Despite the impact of these studies, the choice of engineered mutations can be a source of bias toward previous hypotheses. Moreover, these studies do not collectively cover every residue in the α-synuclein protein, leaving the role of unprobed residues unknown and potentially obscuring additional mechanisms. Perhaps most importantly, the relative sparsity of these data precludes the development of integrative structural models that can be used to identify the key conformational drivers of specific phenotypes, thereby motivating comprehensive mutagenic studies of protein activity.

## Advances

### Deep mutational scanning of α-synuclein

Deep mutational scanning (DMS) is a large-scale mutational data-based method to study protein activity and structure [89,90]. This method has emerged as a powerful tool for systematically quantifying the functional effects of large numbers of protein variants in parallel (Figure 2A). A pooled library encoding missense variants of the protein of interest is introduced into a suitable system, wherein the genotype of the variants is coupled to a measurable phenotype, often either a growth-based fitness defect (Figure 2A–C) or fluorescence-based readout resolved by flow cytometry (Figure 3). Laboratory selection by outgrowth or sorting then enriches the population in variants with perturbed activity, which can be identified using high-throughput DNA sequencing. The relative change in the abundance of each variant before and after selection is analyzed and interpreted to reveal the functional consequences of each amino-acid substitution. The result is generally a high-resolution sequence–activity landscape that can highlight residues/properties essential for structural integrity or activity and uncover previously unappreciated mechanistic features that govern protein behavior. DMS has been employed to identify molecular determinants of protein toxicity and aggregation for a variety of disease-associated proteins, including Aβ, TAR DNA-binding protein 43, and Islet amyloid polypeptide [91–96]. Furthermore, a recent study showed that DMS data have been used to train a neural network model for the aggregation prediction of the proteins from their sequences [97].

**Figure 2:**
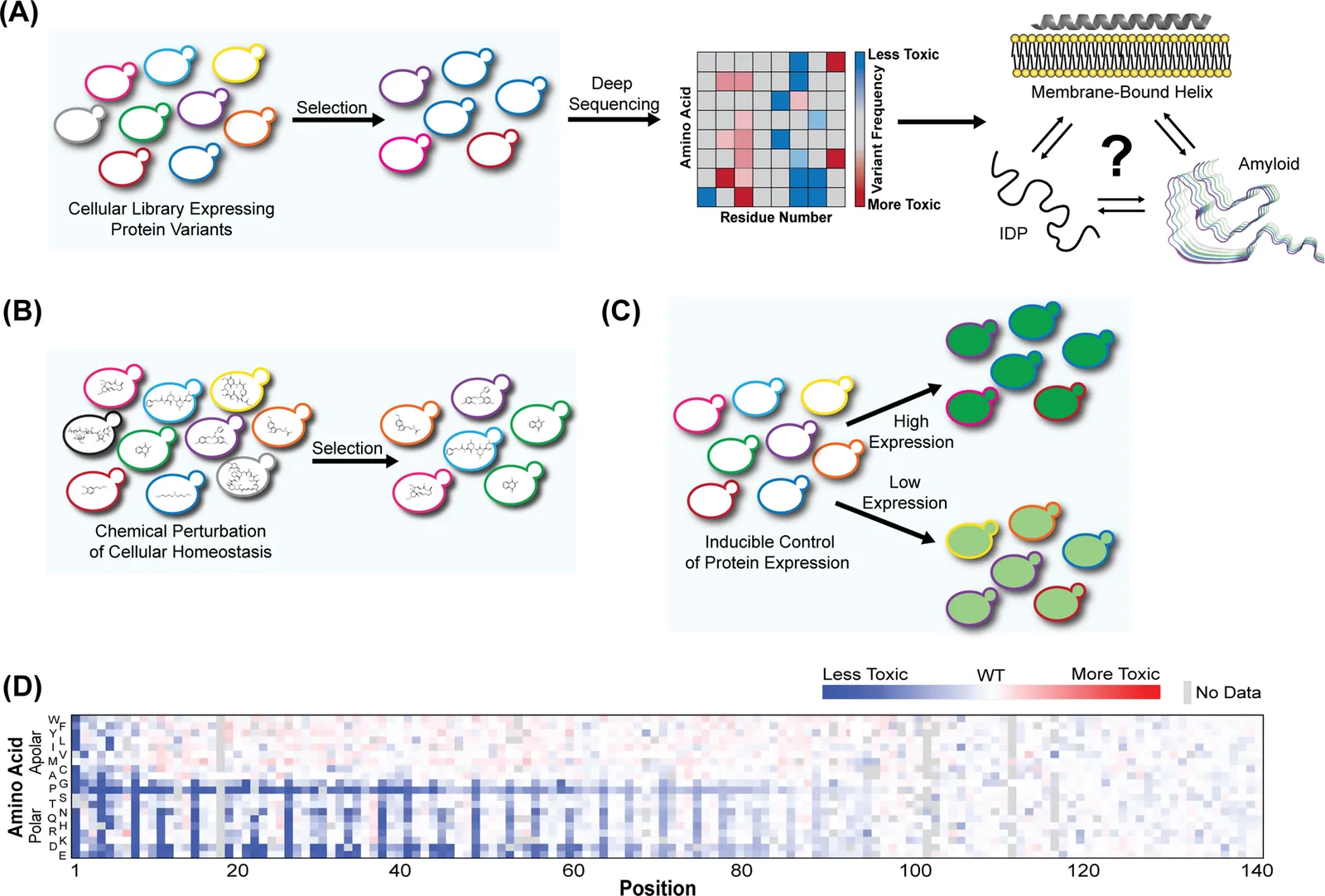
Comprehensive mutagenesis of α-synuclein in yeast (**A**) DMS of α-synuclein to probe the conformational states that drive cellular toxicity [98]. Application of DMS to probe changes in α-synuclein behavior under (**B**) chemical perturbation [99] or (**C**) different protein expression levels [100]. (**D**) Sequence–toxicity landscape of α-synuclein from the experiment diagramed in panel (A). Similar results are obtained for the experiments outlined in panels (B,C).

**Figure 3:**
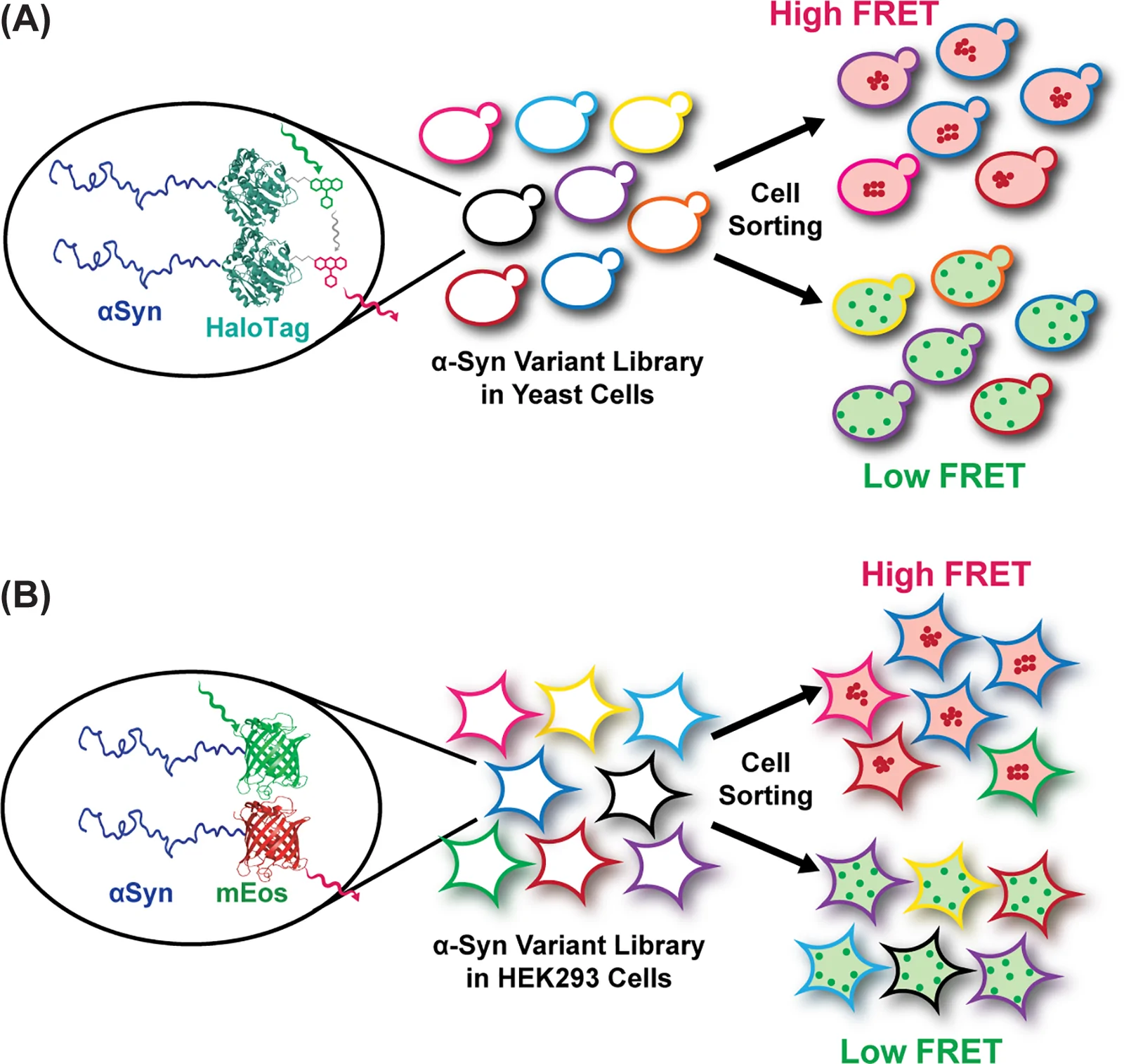
Selection strategies to probe α-synuclein aggregation in high throughput (**A**) HaloTag-based aggregation assay used to probe spontaneous α-synuclein aggregation in yeast [101]. (**B**) mEos-based aggregation assay used to probe seed-mediated aggregation in HEK293T cells [94].

#### DMS identifies the toxic conformational state of α-synuclein in yeast

We sought to exploit DMS to identify conformational states of α-synuclein that are most responsible for the protein’s cytotoxicity, which we expected would clarify the mechanisms of cellular degeneration and inspire strategies for anti-misfolding interventions. To do so, we first needed to identify a cellular model that recapitulates key features of α-synuclein pathology while allowing for the experimental throughput necessary to screen the effects of thousands of mutations in parallel. We therefore focused on *Saccharomyces cerevisiae* (budding yeast), which has been established as a tractable cellular model for interrogating α-synuclein toxicity and aggregation. Though yeast do not natively express any synuclein homologs, heterologous expression of α-synuclein in yeast recapitulates several defining features of its pathobiology in human neurons, including membrane association, aggregation, and dose-dependent cytotoxicity [102,103], making this a powerful system in which to study gain of toxicity; in contrast, because yeast do not participate in synaptic transmission, other models are required for examining loss-of-function mechanisms of pathology. The resulting cytoplasmic puncta sequester endoplasmic reticulum- and Golgi-derived vesicles [104], paralleling the membranous, vesicle-rich architecture of Lewy bodies characterized in PD [48,49,79,105]. Screening of genetic and chemical modifiers of α-synuclein toxicity in yeast has proven fruitful, as several hits have since been validated in neuronal and animal models [43,106–110].

Despite decades of research on this model, it was not entirely clear which of the many conformational states of α-synuclein drive toxicity in yeast; we therefore undertook DMS to address this question [98]. We expressed a comprehensive library of α-synuclein missense variants in yeast and subjected the population to selection by outgrowth. This library covers 99% of the possible single missense variants; the remaining 1% were lost primarily during DNA synthesis. After quantifying the fitness of each variant by deep sequencing, we discovered a consistent pattern of hundreds of mutations that disrupted the ability of α-synuclein to adopt the toxic conformation and restored fitness to α-synuclein-expressing cells (Figure 2A,D).

The pattern of mutations that disrupt α-synuclein toxicity strongly implicated the monomeric, membrane-bound helix as the key toxic species in yeast. For example, proline substitutions reduced toxicity throughout the first 90 residues, indicating that the toxic state is not disordered; otherwise, the sequence should tolerate structure-disrupting proline substitutions. Even more strikingly, introducing new polar residues was sufficient to reduce α-synuclein toxicity but only when incorporated at positions that recurred every 3.67 residues, which closely matches the periodicity of the α-helix. The sensitive positions are hydrophobic in the WT sequence and are predicted to bury into the interior of the lipid bilayer upon membrane binding. To test this prediction, we examined the effect of a subset of these mutations on the membrane-binding affinity. Each variant was titrated with increasing concentrations of model membranes, which induces α-helicity that can be observed by circular dichroism spectroscopy. Consistent with the hypothesis, each of twelve newly designed variants caused consistent effects on both membrane binding and yeast toxicity. This conclusion was further supported by examining the subcellular localization of each variant; substitutions that reduced membrane binding *in vitro* also reduced the appearance of intracellular inclusions, which are known to consist of clustered vesicles tethered by α-synuclein [104]. These results show that α-synuclein toxicity can result from the dysregulation of its native biology [45] and does not necessarily require the transition to a β-sheet-rich aggregate like amyloid fibers. Toxicity of the membrane-bound state of α-synuclein is further supported by (1) functional genomics in yeast, which highlights vesicle trafficking genes as modifiers of α-synuclein toxicity [108] and (2) the vesicle-rich nature of Lewy bodies in Parkinson’s patients [48], wherein α-synuclein seems to participate in the formation of protein–membrane networks that form the pathological hallmarks of the disease.

The sequence–toxicity landscape also showed greater mutational sensitivity toward the N terminus of the amphiphilic helix relative to its C terminus. We considered two possible hypotheses for this relationship: either the helix penetrates the membrane to different depths, or it is variably dynamic along its length. To test this hypothesis, we estimated the burial depth of each of the seven 11-residue membrane-binding repeat segments, using known depth-dependent pseudoenergies associated with positioning each amino acid to specific depths within the lipid bilayer [111]. We found that the effects of each mutation of yeast toxicity best correlated to their predicted effects on membrane binding when the average depth of penetration was approx. 14 Å, and this value did not vary along the length of the helix [99]. Instead, we found that the predicted membrane-binding affinity decreased from N terminus to C terminus, likely leading to increasing dynamics in the membrane-bound state toward the C terminus, which is consistent with results from NMR spectroscopy [14]. These dynamics in the membrane-bound state have a variety of implications. By dynamically dissociating from the membrane, the C-terminal region of the amphiphilic helix could engage nearby membranes [14,112] and promote vesicle clustering [112–115], regulate exocytotic fusion pore dilation [116], and/or nucleate self-association between monomers adsorbed on the membrane surface [117].

To our knowledge, this was the first application of systematic mutagenesis to identify the conformational state of an intrinsically disordered protein that is responsible for a specific cellular phenotype. The comprehensive sequence–toxicity landscape was also sufficient to infer high-resolution features of the structure and dynamics of the bioactive state directly from data collected in living cells. DMS therefore presents great potential for probing complicated folding landscapes directly in complex cellular environments, which we expect can be applied to a variety of other targets as well.

#### Probing environmental sensitivity of α-synuclein folding using DMS

We were surprised that the mutational data so clearly implicated the membrane-bound helix as the single driver of α-synuclein toxicity in yeast, since this state is implicated in the physiological roles of α-synuclein in synaptic vesicle trafficking [118]. Given the broad range of conformations accessible to α-synuclein, which are highly sensitive to local environment, we next asked if changes to the cellular folding environment would either shift the conformational equilibrium and/or sensitize cells to other mechanisms of toxicity. To test this hypothesis, we screened the same α-synuclein variant library under different cellular stresses induced by treatment with a variety of tool compounds that perturb oxidative stress, endoplasmic-reticulum stress, membrane composition, proteasome function, chaperone activity, translation, and autophagy (Figure 2B). Across all of these disparate conditions, the pattern of mutations that disrupt α-synuclein toxicity in yeast remained unchanged, further implicating the membrane-bound state as a driver of toxicity.

Interestingly, we identified one compound that differentially affected the toxicity of some α-synuclein variants to others. Specifically, treatment with rapamycin reduced the disruptive effect of mutations to both the start codon and hydrophobic residues on the membrane-contacting face of the amphiphilic helix. Both sets of mutations dramatically reduce α-synuclein toxicity in the absence of rapamycin, but they are less disruptive in the presence of the compound. Because rapamycin treatment can reduce protein expression levels through its effects on translation and autophagy, these results suggest that cellular toxicity results from an excessive population of the membrane-bound state above a critical threshold. Once the population of membrane-bound α-synuclein is reduced due to lower expression (i.e., due to Met-1 substitution) or reduced membrane affinity (i.e., due to introducing polar groups onto the membrane-contacting face), further reductions in α-synuclein concentration due to rapamycin treatment have less effect on toxicity than on α-synuclein variants with high membrane binding.

To test this hypothesis directly, we repeated the DMS analysis at a variety of different α-synuclein expression levels [100]. We were especially interested in how changes to α-synuclein would affect the sequence–toxicity landscape given that concentration affects not just the population of the membrane state but also the rate of protein aggregation [117,119]. Moreover, changes in concentration can shift the equilibrium between different conformational states, potentially revealing contributions from species that are not evident under other environmental conditions. Importantly, changes in gene dosage are also associated with inherited, early-onset PD [120–123], though the exact mechanism by which gene duplications contribute to pathology remain unclear.

To control α-synuclein expression, we used an inducible promoter, which we titrated with varying concentrations of inducer, resulting in expression levels from minimal to saturating (Figure 2C). These varying cellular conditions create distinct folding environments and selective pressures for DMS. Across all of the expression levels tested, the key features of the sequence–toxicity landscape remained unchanged, including the effects of proline residues, the periodicity in the effects of polar mutations, and the decreasing mutational sensitivity toward the C terminus. As a result, we conclude that the membrane-bound helix is a central driver of toxicity in this system and dominates across various concentration regimes.

Despite the conservation of the sequence–toxicity landscape as a whole, we also observed that specific variants were differentially affected by changes in protein expression level. For example, variants with reduced membrane affinity (e.g., those with negative charges on the membrane interior) showed a distinct sensitivity to concentration compared with variants with WT-like membrane affinity. When α-synuclein expression was reduced from its highest levels to more moderate levels, the toxicity of these variants was more significantly reduced than that of variants with WT-like membrane affinity. We interpret this as a sign that these variants are expressed at a concentration closer to their effective dissociation constant for membrane binding; the population of the bound state is most sensitive to changes in concentration when the protein concentration is close to the dissociation constant. This result also confirmed our explanation for the effects of rapamycin on the toxicity of variants with reduced membrane affinity.

In contrast, when expression levels were further reduced to minimal levels, the same low-affinity variants were less sensitive to changes in concentration than the rest of the population. We speculate that this behavior could result from at least two different mechanisms. First, there could exist a critical concentration of the membrane-bound state below which the cell is insensitive to α-synuclein toxicity; variants with reduced membrane affinity would reach that threshold at cellular expression levels above those of variants with WT-like membrane affinity. Alternatively, unfavorable electrostatic interactions between surface-adsorbed monomers could limit population of the toxic membrane-bound state; variants with reduced membrane affinity might be less affected by this negative cooperativity, thereby reducing their sensitivity to changes in concentration. Regardless of the precise mechanism, our data suggest that regulation of the membrane-bound population is a key determinant of α-synuclein toxicity.

#### DMS of α-synuclein aggregation

Protein aggregates are frequently implicated as key drivers of proteotoxicity in misfolding diseases [124,125]; however, given the conformational diversity of aggregated proteins (see above), it is difficult to determine which type of aggregate is present in any particular system. In this case, the challenges in probing protein conformation in living cells are compounded by the challenges in characterizing aggregate structures to high resolution. DMS offers an opportunity to address this gap as well, provided that protein aggregates can be detected in high throughput across a library of cells expressing different protein variants. Though a variety of strategies exist for doing so [52,126,127], most require co-expression of the same protein variant tagged with two different reporters. This is generally incompatible with DMS because the DNA library is transformed/transfected as a pool, so each cell takes up a variant randomly, which, in the case of co-expression, would result in expression of two different variants that could have confounding effects on phenotype.

To circumvent this challenge, we developed a high-throughput FRET assay based on a clickable protein tag [101]. In this system, a single α-synuclein–HaloTag fusion is labeled with a combination of donor and acceptor fluorophores, thereby generating a FRET pair *in situ* from a single protein construct and avoiding the co-transfection problem (Figure 3A). The relative FRET efficiency then provides a sortable readout that can segregate aggregation-positive from aggregation-negative cells in high throughput. After validating this assay on variants with known effects on aggregation, we extended this approach to a comprehensive variant library expressed in yeast. Unexpectedly, whereas most protein aggregates are believed to be enriched in β-sheet structure, the sequence–aggregation landscape in yeast instead implicated the membrane-bound α-helix as a key driver of aggregation. For example, mutations that disrupted cellular aggregation clustered on the hydrophobic face of the membrane-bound helix and generally reduced membrane binding. Moreover, we did not observe any positions where the effects of mutation correlated strongly with the size of the substituted residue, as one would expect if the mutation reduced aggregation by disrupting a packed protein–protein interface such as a steric zipper [124]. These data indicate that FRET-positive cellular α-synuclein aggregates in yeast are dominated by protein–membrane interactions (i.e., monomers in close proximity on a membrane surface), rather than protein–protein interactions (e.g., amyloids). It is possible that α-synuclein engages in important self-interactions in yeast, but they appear to contribute little to the formation of FRET-positive inclusions.

Though the cellular sequence–aggregation landscape did not show any evidence of amyloid formation, we hypothesized that determinants of cellular aggregation might illuminate unappreciated determinants of membrane-induced amyloid formation. To test this hypothesis, we compared the rate of lipid-induced amyloid formation for variants that had different aggregation scores in yeast. We found that mutations that decrease membrane affinity (e.g., T33N) generally increased the amount of lipid necessary to maximize the rate of amyloid formation, whereas mutations that increase membrane affinity (e.g., T33L) reduce the amount of lipid that is necessary to maximize the rate of amyloid formation. We speculate that these mutation shift equilibrium between the membrane-bound and -unbound populations, thereby changing the rate of concentration-dependent amyloid nucleation and elongation processes [117].

Interestingly, the sequence–aggregation landscape also highlighted glycine residues as aggregation gatekeepers. Substitutions of glycine residues within the hydrophobic NAC region (e.g., Gly68, Gly84, and Gly86), especially with hydrophobic residues or alanine, consistently enhanced aggregation in yeast and promoted membrane-induced amyloid formation at lower lipid-to-protein ratios *in vitro*. We speculate that the conformational flexibility of these glycine residues decreases the probability that the protein can adopt aggregation-competent structures, thereby slowing the aggregation process. These data show that a single DMS experiment can reveal sequence determinants that affect multiple different molecular processes (i.e., cellular aggregation versus membrane-induced amyloidogenesis).

Parallel efforts by the Diamond and Joachimiak labs exploited DMS to understand the molecular determinants of a distinct aggregation process in a different cellular environment [94]. Specifically, they applied DMS to α-synuclein aggregation in HEK293 cells, which can be induced by transduction with preformed amyloid seeds. By using a photoconvertible fluorescent protein tag (mEos), they were able to generate a FRET pair *in situ* that identifies cells expressing α-synuclein variants that disrupt the aggregation induced by the exogenous seeds (Figure 3B). Remarkably, the resulting sequence–aggregation landscape differs dramatically than that obtained for spontaneous aggregation in yeast. In the mammalian system, mutations within residues 67–78 strongly hindered seeded aggregation, and mutational sensitivity extended across much of the known amyloid-prone core, including the hydrophobic NAC domain. The screen also identified aggregation enhancers outside the fibril core, most notably E13K, indicating that regions beyond the canonical amyloid core contribute to seed propagation. Follow-up truncational studies, cross-linking mass spectrometry, and NMR spectroscopy supported a model in which transient local structure in the N terminus inhibits aggregation by limiting productive engagement with the amyloid-prone core. They were further able to apply these insights to develop HEK293 lines with tunable sensitivity to transduction by seeding-competent aggregates, which have implications as cellular biosensors of prion-like activity.

The dramatic differences in the fitness landscapes for mutagenesis in yeast versus HEK293 cells highlight two important considerations for the design of DMS analyses. First, the cellular environment plays a key role in shaping the conformational landscape. These cell types will differ both in their proteomes and lipidomes, and both proteins and lipids have been shown to alter the conformational landscape of α-synuclein. This is an important consideration given that PD generally involves selective degeneration of dopaminergic neurons, and the molecular origins of this cell-type selectivity remain unclear. Neither yeast nor HEK293 fully recapitulate key features of α-synuclein behavior in neurons (e.g., high levels of endogenous expression, participation in synaptic transmission), so it is likely that neurons would impose additional constraints on the conformational landscape that are yet unknown. Second, these experiments inherently probe different aggregation processes; experiments in yeast focus on spontaneous toxic species, whereas experiments in HEK293 profile cellular aggregates templated by exogenous amyloids. These are two different pathways for aggregation, and it is perhaps unsurprising that they would have different molecular determinants. Given the remarkable conformational diversity of α-synuclein, additional experiments would be necessary to map the sequence determinants of other aggregation processes (e.g., oligomer formation or liquid–liquid phase separation).

## Conclusions

Herein, we have summarized how DMS has been used to study the sequence determinants of α-synuclein toxicity and aggregation in cells. In yeast toxicity studies, the dominant determinant is membrane affinity encoded by the N-terminal amphiphilic helix. Altered folding environments and expression levels modulate the population of this state, but the key structure that mediates toxicity remains unchanged. Aggregation-coupled selections extend this framework, thereby distinguishing the determinants of toxicity versus aggregation. Spontaneous inclusions in yeast are dominated by membrane-associated assemblies, whereas seeded aggregation in mammalian cells requires amyloid-core compatibility and regulatory interactions outside the core.

These studies argue for an ensemble-based view of α-synuclein pathology. Membrane binding, amyloid formation, oligomerization, and local flexibility can each contribute to pathology under different selections and cellular contexts. DMS can identify which structural states are relevant to a specific phenotype, which can then be prioritized for further investigation. Future studies can build on this capacity by analyzing epistasis between mutations, exploring a wider variety of cellular models, integrating post-translational modifications, profiling disease-or patient-derived seeds, and leveraging DMS datasets to drive computational models of the conformational landscape.

## Perspectives

- α-Synuclein misfolding is central to synucleinopathies like PD, but the protein populates multiple, interconverting conformational states, and it is unclear which species drive toxicity and aggregation in cells, complicating the design of therapeutic interventions.
- By quantifying the effects of a comprehensive set of missense variants on α-synuclein activity, DMS can reveal the structural basis for different phenotypes, which has implicated an extended, membrane-bound amphiphilic helix as the dominant driver of both toxicity and aggregation in yeast.
- Understanding the structural basis for the complete portfolio of α-synuclein behaviors will require extending this approach to a wider variety of cellular systems, such as neuronal and patient-derived systems, as well as diversifying the approaches used to select and analyze variants of interest.

## Open Access

Open access for this article is provided via our Subscribe to Open programme, made possible through the support of our subscribing institutions. Learn more here: https://portlandpress.com/pages/open_access_options_and_prices#conditional.

## Abbreviations

DMS: deep mutational scanning
FRET: Förster resonance energy transfer
NAC: non-amyloid β component
NMR: nuclear magnetic resonance
PD: Parkinson’s disease
